# Mesonephric-Like Carcinosarcoma of the Ovary Associated with Low-Grade Serous Carcinoma: A Case Report

**DOI:** 10.3390/diagnostics11050827

**Published:** 2021-05-03

**Authors:** Antonio d’Amati, Federica Pezzuto, Gabriella Serio, Andrea Marzullo, Francesco Fortarezza, Teresa Lettini, Gerardo Cazzato, Gennaro Cormio, Leonardo Resta

**Affiliations:** 1 Department of Emergency and Organ Transplantation, Section of Pathology, University of Bari “Aldo Moro”, 70124 Bari, Italy; antonio.damati@uniba.it (A.d.); andrea.marzullo@uniba.it (A.M.); lettinit@yahoo.com (T.L.); gerycazzato@hotmail.it (G.C.); leonardo.resta@uniba.it (L.R.); 2 Department of Cardiac, Thoracic, Vascular Sciences and Public Health, University of Padova, 35121 Padova, Italy; federica.pezzuto@phd.unipd.it (F.P.); francescofortarezza.md@gmail.com (F.F.); 3 Department of Biomedical Sciences and Medical Oncology, University of Bari “Aldo Moro”, 70124 Bari, Italy; gennaro.cormio@uniba.it

**Keywords:** pathology, gynecological pathology, ovarian cancer, mesonephric adenocarcinoma, mesonephric-like carcinoma, carcinosarcoma, mesonephric carcinosarcoma

## Abstract

Mesonephric adenocarcinomas are rare tumors of the female genital tract, thought to arise from embryonic mesonephric remnants, primarily in the cervix and vagina. Conversely, endometrial and ovarian mesonephric adenocarcinomas may have a different pathogenesis, probably originating from transdifferentiated Müllerian carcinomas, as demonstrated by the association of these neoplasms with endometriosis and ovarian serous tumors. For this reason, in the endometrium and in the ovary, they are defined as “mesonephric-like adenocarcinomas”. Some cases of mesonephric carcinomas of the female genital tract have been reported to show a sarcomatous component and have been defined as “mesonephric carcinosarcomas”, characterized by poor prognosis and high metastatic behavior, but this entity has never been described in the ovary. The case herein presented is of a 74-year-old female with abdominal discomfort and a complex ovarian mass. Histological and immunohistochemical analysis showed features of ovarian mesonephric-like carcinoma combined with a low-grade serous component, in support of the theory of a Müllerian origin of these neoplasms. The tumor also revealed foci of chondrosarcomatous differentiation, never before reported in the ovary, showing a similar immunohistochemical profile to the mesonephric-like elements. This work thus describes the first reported case of ovarian mesonephric-like carcinosarcoma.

## 1. Introduction

Mesonephric adenocarcinomas are very rare malignancies of the female genital tract, arising in the uterine cervix or, less frequently, in the uterine body, in the vagina or in the ovary [1,2,3]. These tumors are thought to originate from mesonephric (Wolffian) remnants, frequently observable in the walls of the uterine cervix, or more rarely in the vagina, in the uterine body, in the broad ligaments or in the ovarian hilum [4,5]. While the origin of cervical and vaginal mesonephric adenocarcinomas from Wolffian remnants seems to be widely accepted, the pathogenesis of endometrial and ovarian mesonephric carcinomas remains uncertain. In fact, in the fifth edition of the WHO Classification for Female Genital Tumors, they are defined as “mesonephric-like adenocarcinomas” [6]. According to some authors, these tumors may arise from Müllerian carcinomas with mesonephric transdifferentiation, as supported by the frequent association in the ovary of mesonephric-like carcinomas with endometriosis, serous cystadenomas, serous borderline tumors and low-grade serous carcinomas [1,7,8,9]. Moreover, ovarian mesonephric-like adenocarcinomas associated with serous borderline tumors or low-grade serous carcinomas have mutations of KRAS or NRAS in both elements [8,9,10,11]. Some cases of vaginal or uterine mesonephric carcinomas reported in literature show a malignant mesenchymal component, recently considered as a metaplastic change [12,13,14,15]. These extremely rare tumors have been defined as vaginal, cervical or endometrial mesonephric carcinosarcomas but, until now, a sarcomatous component in ovarian mesonephric-like adenocarcinomas has never been reported in literature, and the ovarian mesonephric-like carcinosarcoma is not included in the last WHO Classification for Female Genital Tumors [6]. We report herein the first case of ovarian mesonephric-like carcinosarcoma.

## 2. Case Presentation

### 2.1. Clinical History

A 74-year-old female, with a history of abdominal discomfort, was referred in August 2020 to the gynecologic oncology unit of Bari Policlinico hospital by her gynecologist for a complex ovarian mass. In 1973 she underwent right salpingo-oophorectomy for an ovarian serous cystadenoma. All four of the patient’s brothers had a history of cancer (pancreatic ductal adenocarcinoma, glioblastoma, gastric adenocarcinoma and lung adenocarcinoma). In our patient, transvaginal echography showed a solid-cystic tumor in the left ovary, with a maximum diameter of 5 cm. CT scan confirmed the ovarian mass, also revealing evidence of peritoneal carcinomatosis and suspected omental metastases. Exploratory laparoscopy, with a peritoneal nodule biopsy, was performed for histological diagnosis. Pathology results demonstrated a peritoneal localization of a malignant epithelial neoplasm, with necrosis and a mixed glandular and solid pattern, suspected to be an ovarian poorly differentiated endometrioid adenocarcinoma. After diagnosis, the patient underwent 12 cycles of neoadjuvant chemotherapy (carboplatin and paclitaxel), with a partial regression of the tumor. Subsequently, she underwent hysterectomy with left salpingo-oophorectomy, large-bowel segmental resection, omentectomy and resection of two peritoneal nodules.

### 2.2. Pathological Findings

**Figure 1:**
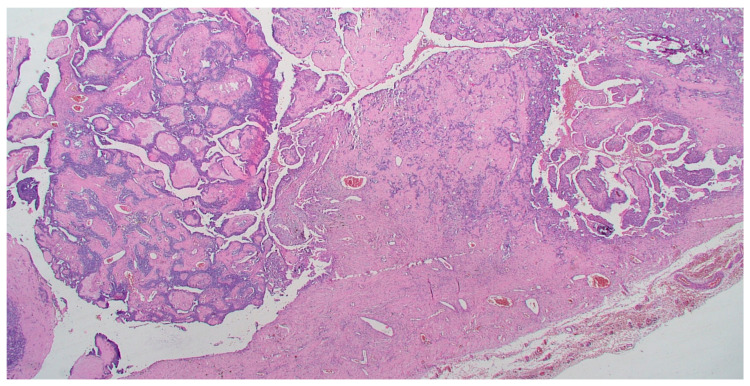
Transition from serous borderline tumor to low-grade serous and mesonephric-like adenocarcinoma. (HE, 40×).

**Figure 2:**
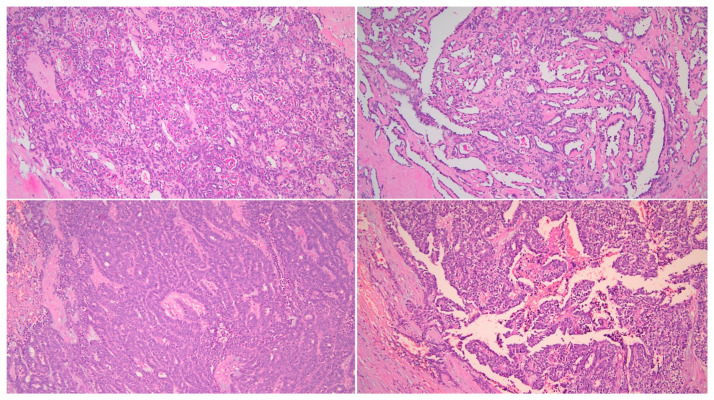
Mesonephric-like adenocarcinoma showing different patterns. (HE, 200×).

**Figure 3:**
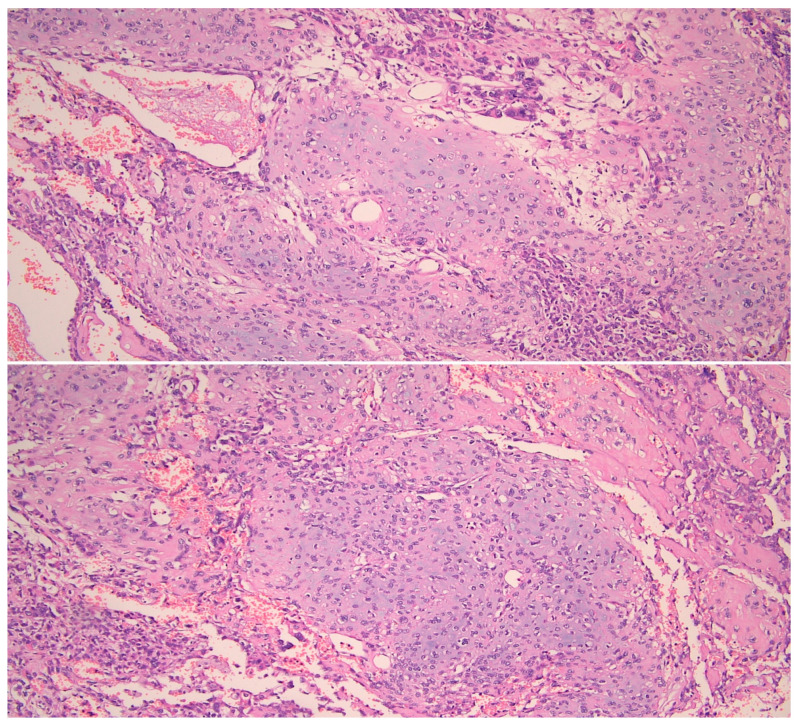
Chondrosarcomatous component intermingled with carcinomatous neoplastic cells. (HE, 200×).

On gross pathology examination, the left ovary measured 4.5 × 3 × 2 cm, showing an intact, smooth outer surface. The cut section revealed a small unilocular cyst with an adjacent large, solid mass. The left Fallopian tube and the uterus were grossly normal. The omentum presented multiple nodules, the largest of which measured 4 cm. The resected large bowel segment showed a 5 cm nodule on the serosal surface. Histologically, the left ovary showed a serous cystadenoma, undergoing progressive transition to a serous borderline tumor and invasive carcinoma (Figure 1). The invasive part showed focal histological features of low-grade serous carcinoma (10%), but was mainly composed of a mesonephric-like adenocarcinoma (85%), showing tubular, glandular (pseudoendometrioid), sex cord-like and solid patterns (Figure 2). The tubular structures lumens contained typical eosinophilic colloid-like secretions. The nuclei were monomorphic, predominantly with vesicular chromatin and inconspicuous nucleoli. Focal lymphovascular invasion was present. There were no squamous or mucinous elements. However, a part of the neoplasm showed a chondrosarcomatous component (5%), intermingled with carcinomatous elements (Figure 3). The chondrosarcomatous component was observed also in the intestinal nodule, along with glandular and solid carcinomatous elements, infiltrating from the visceral peritoneum into the pericolic fat. The omental and peritoneal nodules revealed the same histological features as the ovarian mesonephric-like adenocarcinoma, without sarcomatous differentiation. 

**Figure 4:**
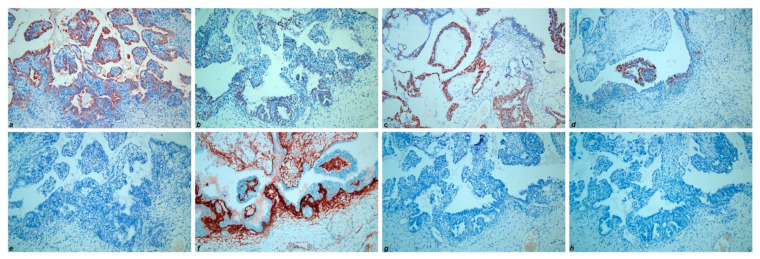
Immunohistochemical results of low-grade serous carcinoma component: (**a**) CK7; (**b**) PAX8; (**c**) ER; (**d**) PR; (**e**) Calretinin; (**f**) CD10; (**g**) TTF-1; (**h**) GATA-3. (200×).

**Figure 5:**
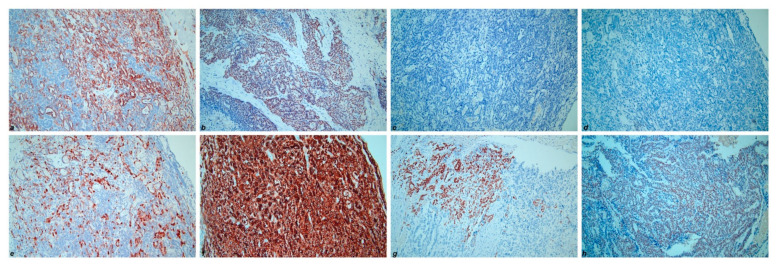
Immunohistochemical results of mesonephric-like adenocarcinoma component: (**a**) CK7; (**b**) PAX8; (**c**) ER; (**d**) PR; (**e**) Calretinin; (**f**) CD10; (**g**) TTF-1; (**h**) GATA-3. (200×).

**Figure 6:**
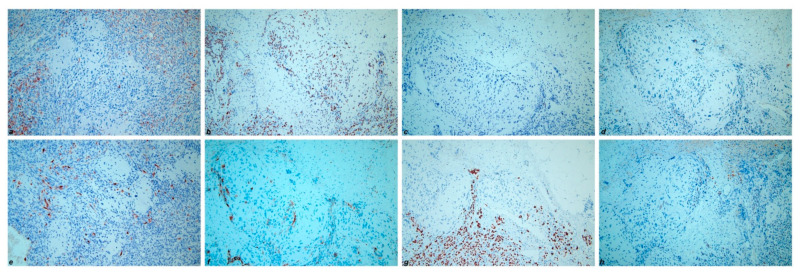
Immunohistochemical results of chondrosarcomatous component: (**a**) CK7; (**b**) PAX8; (**c**) ER; (**d**) PR; (**e**) Calretinin; (**f**) CD10; (**g**) TTF-1; (**h**) GATA-3. (200×).

Immunohistochemically, the low-grade serous component showed positivity for CK7 (cytokeratin 7), PAX8 (paired box gene 8), ER (estrogen receptor), PR (progesterone receptor) and complete negativity for Calretinin, CD10, TTF-1 (transcriptional thyroid factor 1) and GATA-3 (GATA binding protein 3) (Figure 4). On the contrary, the mesonephric-like component revealed negativity for both ER and PR, along with positivity for CK7, PAX8, Calretinin, CD 10 (luminal staining in tubular pattern areas), TTF-1 and GATA-3 (Figure 5). Furthermore, 20% of tumoral cells showed a strong membranous staining for PD-L1 (CC23 clone). The chondrosarcomatous component showed the same immunoreactivity as the mesonephric-like component, even though more focal and weaker (Figure 6). The immunohistochemical results of the different tumor components are resumed in Table 1. All three components revealed wild-type p53 expression. The Ki67 proliferative index showed a heterogeneous expression pattern, with variable scores in different areas (from 5% to 80%). The final FIGO stage was IIIC.

## 3. Discussion

Mesonephric adenocarcinomas are very rare tumors of the female genital tract. They usually arise in the uterine cervix, where it is more common to observe mesonephric remnants, but may originate also in the vagina, uterine corpus and ovary [1,2,3]. These tumors are believed to arise from embryonic remnants of Wolffian ducts, that incompletely regress during normal female embryological development. In the early phases of embryological development, a pair of mesonephric (Wolffian) ducts, adjacent to the paramesonephric (Müllerian) ducts, link the developing kidney to the cloaca. In the male, the anti-Müllerian hormone secreted by Sertoli cells induces paramesonephric (Müllerian) duct regression, while the mesonephric (Wolffian) ducts develop into testis efferent ducts, epididymis, vas deferens and seminal vesicles. In the female, the absence of the anti-Müllerian hormone leads to regression of the mesonephric (Wolffian) ducts, while the paramesonephric (Müllerian) ducts fuse and develop into fallopian tubes, the uterus and upper part of the vaginal wall. Remnants of mesonephric (Wolffian) ducts may persist in females along the lateral walls of the vagina, cervix, uterine corpus, broad ligaments and ovarian hilum. These mesonephric vestiges are thought to be the origin of mesonephric carcinomas of the female genital tract because they may expand into hyperplastic proliferations and, in some cases, give rise to malignant neoplasms [5,16]. However, while the origin of vaginal and cervical mesonephric carcinomas from Wolffian remnants has been demonstrated and accepted, the endometrial and ovarian counterparts may have a different pathogenesis. Different studies have suggested that endometrial and ovarian mesonephric carcinomas, or at least some of them, may arise from Müllerian carcinomas, through a mesonephric transdifferentiation process. For this reason, these tumors in the endometrium and ovary are defined as mesonephric-like adenocarcinomas. The theory of a Müllerian origin of ovarian mesonephric-like carcinomas is supported by the common association of these neoplasms with endometriosis, serous cystadenomas and adenofibromas, serous borderline tumors and low-grade serous carcinomas [1,7,8,9]. Furthermore, ovarian mesonephric-like adenocarcinomas associated to serous ovarian neoplasms share the same KRAS or NRAS mutations in both components. Some tumors also showed PIK3CA mutations, gain of 1q and loss of 1p [8,9,10,11]. 

The histological features of these tumors are peculiar. They usually show an admixture of different architectural patterns, such as tubular, glandular (variously sized), papillary, sex cord-like, slit-like, retiform and solid. The tubular and glandular patterns are the most common and often contain typical intraluminal secretions, composed of eosinophilic colloid-like material. The tumor cells show a variable shape and size, usually with a scanty eosinophilic cytoplasm and monotonous hyperchromic or vesicular nuclei [5,16]. The case herein presented showed a predominant carcinomatous component with histological features of mesonephric-like adenocarcinoma, associated with a low-grade serous carcinoma component, arising from an ovarian serous cystadenoma. These interesting histological features are in accordance with other cases in literature, demonstrating the association of mesonephric-like adenocarcinomas with other ovarian neoplasms, and may offer further proof of the possible Müllerian origin of these tumors.

Moreover, part of the tumor showed, both in the ovary and in the intestinal nodule, a mesenchymal malignant component consistent with a chondrosarcomatous appearance. In literature, some cases of uterine or vaginal mesonephric adenocarcinomas have been reported, associated with a sarcomatous component [12,13,14,15]. Neoplasms that contain both epithelial and mesenchymal malignant elements are defined as carcinosarcomas (previously known as malignant mixed Müllerian tumors), even though the mesenchymal component probably represents a metaplastic change of the epithelial component. Nevertheless, at the current state of the art, no cases of ovarian mesonephric-like adenocarcinomas have been found associated with a malignant mesenchymal component, before the case presented in this report. The immunophenotype of ovarian mesonephric-like adenocarcinomas, like the morphology, is similar to mesonephric adenocarcinomas of the female genital tract. They are usually positive for PAX8, negative for hormone receptors (ER and PR) and have a “wild-type” immunoreactivity for p53. Furthermore, they may show positivity for calretinin (nuclear and cytoplasmic staining), CD10 (luminal staining in tubular and glandular structures), TTF-1 and GATA-3 (both nuclear, usually focal) [16,17,18]. Our case showed a similar immunostaining profile in the mesonephric-like component, with positivity for CK7, Calretinin, CD10, TTF-1, GATA-3 and PAX8 and negativity for ER and PR. We also tested PD-L1 immunoreactivity, to see whether to attempt an immunotherapy treatment protocol along with adjuvant chemotherapy. By contrast, the low-grade serous part revealed positivity for ER and PR, along with negativity for Calretinin, CD10, TTF-1 and GATA-3, confirming the morphological appearance. Interestingly, the chondrosarcomatous component showed the same immunophenotype as the mesonephric-like component. This observation, never before reported in literature, may indicate a further step in the transdifferentiation process of these neoplasms, from Müllerian neoplasms to a mesonephric-like morphology and, finally, to a mesenchymal-like morphology. Another point of interest of this case is the focal CK7 positivity in the sarcomatous component. This finding suggests that the mesenchymal morphology may represent a metaplastic change of the carcinomatous part, rather than a true sarcomatous combined neoplasm, and also suggests that carcinosarcomas of the female genital tract are probably metaplastic carcinomas in which the mesenchymal part retains morphological and immunohistochemical features of the epithelial component.

## 4. Conclusions

Additional studies with larger numbers of cases would be fundamental in order to determine the real origin of these neoplasms and the molecular processes that lead Müllerian neoplasms to transdifferentiate into mesonephric-like carcinomas and, as reported for the first time in this work, into a sarcomatous morphology.

The case herein reported sheds light on the similar histological features and behavior of these neoplasms in all sites of the female genital tract, demonstrating that ovarian mesonephric-like adenocarcinomas may also have a malignant mesenchymal component, thus being definable as “ovarian mesonephric-like carcinosarcoma”. Therefore, we can suppose that it is only because of the extreme rarity of ovarian mesonephric-like adenocarcinomas that this sarcomatous transdifferentiation has never previously been reported. 

Finally, the relation between the sarcomatous component and prognosis will need to be investigated in further studies, and also the prognosis of ovarian mesonephric-like adenocarcinomas, in order to obtain evidence-based treatment guidelines for patients affected by this kind of rare neoplasm.

## Figures and Tables

**Table 1:** Immunohistochemical results of the different tumor components (legend: + = positive immunohistochemical result, - = negative immunohistochemical result).

	Low-Grade Serous Component	Mesonephric-Like Component	Sarcomatous Component
CK7	+	+	+
PAX8	+	+	+
ER	+	-	-
PR	+	-	-
Calretinin	-	+	+
CD10	-	+	+
TTF-1	-	+	+
GATA-3	-	+	+
